# Supplementary material for: Adsorption of Chromium, Copper, Lead, Selenium, and Zinc ions into ecofriendly synthesized magnetic iron nanoparticles
Source: PLoS One. 2023 Oct 18;18(10):e0289709. doi: 10.1371/journal.pone.0289709 (PMC10584173; doi:10.1371/journal.pone.0289709)
Supplement: Attachment — Submitted filename: Review PLOS ONE.pdf [file pone.0289709.s003.pdf]

Reviewer Comments

PLOS ONE

PONE-D-23-05787R1

Title: Adsorption of Chromium, Copper, Lead, Selenium, and Zinc Ions into Ecofriendly Synthesized Magnetic Iron Nanoparticles"

Comments

This manuscript evaluates the ability of synthesized sorbents (Magnetic Iron Nanoparticles, Fe-NPs) derived from the peel extracts of banana, pomegranate, opuntia, orange, potato, and onion for efficient removal of Pb, Se, Cu, Zn, and Cr from aqueous solutions. The authors claimed that the prepared Fe-NPs adsorbents were effective in removing the studied heavy metals. However, the following technical aspects of the present work are lacking and were note appropriately addressed:

1) Abstract is lacking some numeric data.
2) The powerful properties of the newly prepared magnetic Fe-NPs should be clearly demonstrated and linked to the conclusions.
3) Fig (1) is not referred to in the text.
4) The particle size and the BET surface areas of magnetic Fe-NPs should be presented.
5) Standard errors should be added to Figs.8&9.
6) Please compare the adsorption capacity of the present work with other adsorbents reported in literature and provide a comparison table in the manuscript.
7) In conclusions section, the authors should clearly present conclusions of the circumstances under which their approach would be most effective.

Recommendation

Major revision
