# Supplementary material for: Adsorption of Chromium, Copper, Lead, Selenium, and Zinc ions into ecofriendly synthesized magnetic iron nanoparticles
Source: PLoS One. 2023 Oct 18;18(10):e0289709. doi: 10.1371/journal.pone.0289709 (PMC10584173; doi:10.1371/journal.pone.0289709)
Supplement: Attachment — Submitted filename: The manuscript comment.docx [file pone.0289709.s001.docx]

The manuscript “Adsorption of Chromium, Copper, Lead, Selenium, and Zinc Ions into Ecofriendly Synthesized Magnetic Iron Nanoparticles” does not meet the minimum required scientific standard for publication in PLOS ONE. Although the work is very specific, the synthesis of magnetic Iron nanoparticles using eco-friendly methods is well studied in previous publications. The following major comments can be clarified to improve acceptability. See the comments below:

1. Section 1. The author suggests “The particle size, magnetic properties, and morphology of Fe-NPs depend on the conditions of the material” but fails to prove the statement in the characterization. SEM and /or TEM are strongly recommended for morphology and particle size measurement.
2. Section 2.2 “The production of extracts and the synthesis of Fe-NPs were carried out according to Niraimathee et al [19]”. Include the method and modification done.
3. Section 2.3 – it is not clear whether the adsorption of heavy metals was simultaneous or individual.
4. Section 3.1.2 - The inclusion of SEM results will show how morphology changes with different modifications.
5. Section 3.1.4 - “The change in the position of the absorption peak of the iron colloidal nanoparticles may be due to the change in the size of the colloidal nanoparticles [18]”. TEM results will support the changes in particle size.
6. Section 3.1.5 – The author should include EDX spectra after adsorption.
7. The author should include TGA results to assess the thermal stability of adsorbents.
8. Section 3.2.1 and 3.2.2 – Explain a significant drop in copper adsorption in figure 7 (17.26 & 20.00% respectively).
9. Can the authors compare the adsorption capacity of the adsorbents with other reported similar adsorbents?
10. The author should include pH and temperature studies as heavy metals behave differently in solution.
11. The authors should give some more information on the mechanism of interaction of the heavy metals with adsorbents, which could be in terms of the ionic forms present and the surface of the powder since they are pH dependent. The author should include the isotherm model, kinetics and thermodynamics to understand the adsorption mechanism.
12. Are these results reproducible with different batches of the adsorbents? Have you done the error analysis? Is there any effect on the size of the powder?
13. A regeneration study is important to evaluate the cost-effectiveness of the adsorbent
