# Supplementary material for: Adsorption of Chromium, Copper, Lead, Selenium, and Zinc ions into ecofriendly synthesized magnetic iron nanoparticles
Source: PLoS One. 2023 Oct 18;18(10):e0289709. doi: 10.1371/journal.pone.0289709 (PMC10584173; doi:10.1371/journal.pone.0289709)
Supplement: Attachment — Submitted filename: Respond to Reviewers.docx [file pone.0289709.s002.docx]

**PONE-D-23-05787**

**Adsorption of Chromium, Copper, Lead, Selenium, and Zinc Ions into Ecofriendly Synthesized Magnetic Iron Nanoparticles**

| **Academic Editor's comments:** | | |
| --- | --- | --- |
| **Provide a conclusion in the abstract** | **(Done)** | |
| **2) pH is one of the most critical parameters influencing the adsorption of contaminants as it affects the surface chemistry and ionization of species among other things. In the current paper, various metal ions were studies and these may have different optimum pH for the removal by the nanoparticles** | **Done**  **2.3.4 Effect of pH** | |
| **3) Also, it is important that experiments are reproducible. How many replicates were used by the authors?** | **(Three time) Done** | |
| **4) SEM and TEM analysis must be included in the** | **3.1.6 Scanning Electron Microscopy (SEM) and TEM not applicable** | |
| **5) Section 2.2 must reference Figure 1.** | **Done** | |
| **6) Section 2.3, describe the filter used. Is there any chance that it might have participated in the adsorption? Also considering that low concentrations of pollutants were investigated?** | **Done** | |
| **7) Standardize the units throughout the manuscript.** | **Done** | |
| **8) Sections 2.3.1, 2.3.2 and 2.3.3 can all be combined into one section.** | **Done** | |
| **9) Section 3.1.2. Delete the repeating sentence** | **Done** | |
| **10) The mechanism of the adsorption process must be explained/ isotherm, kinetic** | **Done, 2.4 Batch studies using adsorbent** | |
| **11) Kinetic models and adsorption isotherms must be used to interpret the data.** | **Done**  **2.5 Kinetic Study** | |
| **Reviewer #1:** | | |
| **1. Section 1. The author suggests “The particle size, magnetic properties, and morphology of Fe-NPs depend on the conditions of the material” but fails to prove the statement in the characterization. SEM and /or TEM are strongly recommended for morphology and particle size measurement.** | | **3.1.6 Scanning Electron Microscopy (SEM) and TEM not applicable** |
| **2. Section 2.2 “The production of extracts and the synthesis of Fe-NPs were carried out according to Niraimathee et al [19]”. Include the method and modification** | | **done.** |
| **3. Section 2.3 – it is not clear whether the adsorption of heavy metals was simultaneous or individual.** | | **All metals dissolved in same solution (simultaneous)** |
| **4. Section 3.1.2 - The inclusion of SEM results will show how morphology changes with different modifications.** | | **Done**  **3.1.6 Scanning Electron microscope** |
| **5. Section 3.1.4 - “The change in the position of the absorption peak of the iron colloidal nanoparticles may be due to the change in the size of the colloidal nanoparticles [18]”. TEM results will support the changes in particle size.** | | **TEM, not available** |
| **6. Section 3.1.5 – The author should if include EDX spectra after adsorption.** | | **The EDX for materials FeNPs before done but after absorption Not, available** |
| **7. The author should include TGA results to assess the thermal stability of adsorbents.** | | **TGA,Not,available** |
| **8. Section 3.2.1 and 3.2.2 – Explain a significant drop in copper adsorption in figure 7 (17.26 & 20.00%respectively)** | | **The removal efficiency opuntia is low due to in the vacant site for adsorption and free electrons for the degradation process is very low other than adsorbents.** |
| **9. Can the authors compare the adsorption capacity of the adsorbents with other reported similar adsorbents?** | | **Table 6: Adsorption efficiencies of some selected adsorbents** |
| **10. The author should include pH and temperature studies as heavy metals behave differently in solution** | | **2.3.4 Effect of pH, and temperature not applicable** |
| **11. The authors should give some more information on the mechanism of interaction of the heavy metals with adsorbents, which could be in terms of the ionic forms present and the surface of the powder since they are pH dependent. The author should include the isotherm model, kinetics and thermodynamics to understand the adsorption mechanism.** | | **2.5 Kinetic Study**   2.4 **Batch studies using adsorbent**   **2.3.4 Effect of pH** |
| **12. Are these results reproducible with different batches of the adsorbents?** | | **Done, Three time** |
| **Have you done the error analysis? Is there any effect on the size of the powder?** | | **Not applicable** |
| **13. A regeneration study is important to evaluate the cost-effectiveness of the adsorbents** | | **Done**  **3.6 Cost of adsorbents** |
