# Supplementary material for: Adsorption of Chromium, Copper, Lead, Selenium, and Zinc ions into ecofriendly synthesized magnetic iron nanoparticles
Source: PLoS One. 2023 Oct 18;18(10):e0289709. doi: 10.1371/journal.pone.0289709 (PMC10584173; doi:10.1371/journal.pone.0289709)
Supplement: Attachment — Submitted filename: Response to Reviewers.docx [file pone.0289709.s004.docx]

**PONE-D-23-05787**

**Adsorption of Chromium, Copper, Lead, Selenium, and Zinc Ions into Ecofriendly Synthesized Magnetic Iron Nanoparticles**

| **Academic Editor's comments:** | |
| --- | --- |
| **1) Abstract is lacking some numeric data.** | **Done**  The sequence of removal efficiencies of nanoparticles as banana > potato > orange > onion > pomegranate > opuntia for removal heavy metals. The maximum adsorption capacity obtained from the equilibrium studies was in the following order: potato> orange>, onion> banana> pomegranate> opuntia. The pH 6.0 is more efficient in this experiment. The model that best fitted for metal adsorption was Freundlich, and pseudo-second-order. The FeNPs_3_ can be recycled 5.72 times to adsorb Cu, Cr, Pb, Se, and Zn. The cost for 100 g of adsorbents is 3.980 L.E**.** |
| **2) The powerful properties of the newly prepared magnetic Fe-NPs should be clearly demonstrated and linked to the conclusions.** | **Done**  The optimum condition for removal Cu, Cr, Pb, Se, and Zn by using nanoparticle are pH 6.0, stirring rate at 150 rpm, at room temperature 25 ^°^C, dose 0.4, initial concentrations of 3.0 mg/L, and contact time 45 min. The banana, potato, orange, onion, and pomegranate were high efficiency but the opuntia was lower efficiency. The model that best fitted for metal adsorption was Freundlich, and pseudo-second-order. The FeNPs_3_ can be recycled 5.72 times to adsorb Cu, Cr, Pb, Se, and Zn. The cost for 100 g of adsorbents is 3.980 L.E. |
| **3) Fig (1) is not referred to in the text** | **Done**  Figure 1 describes the procedures used for preparation of nanoparticles used in this study. |
| **4) The particle size and the BET surface areas of magnetic Fe-NPs should be presented.** | **Dear Valued Reviewer:**  **The particles size determined by Scanning Electron Microscopy (SEM) and the BET instrument is not applicable.** |
| **5) Standard errors should be added to Figs.8&9** | **Done** |
| **6) Please compare the adsorption capacity of the present work with other adsorbents** | **Done**  **Table 6: Adsorption efficiencies of some selected adsorbents** |
| **7) In conclusions section, the authors should clearly present conclusions of the circumstances under which their approach would be most effective.** | **Done**  The optimum condition for removal Cu, Cr, Pb, Se, and Zn by using nanoparticle are pH 6.0, stirring rate at 150 rpm, at room temperature 25 ^°^C, dose 0.4, initial concentrations of 3.0 mg/L, and contact time 45 min. The banana, potato, orange, onion, and pomegranate were high efficiency but the opuntia was lower efficiency. The model that best fitted for metal adsorption was Freundlich, and pseudo-second-order. The FeNPs_3_ can be recycled 5.72 times to adsorb Cu, Cr, Pb, Se, and Zn. The cost for 100 g of adsorbents is 3.980 L.E. |

|  | Pb | Cu | Cr | Zn | Se |
| --- | --- | --- | --- | --- | --- |
| banana | 46.5167 | 45.2667 | 20 | 43.8333 | 43.4 |
| potato | 93.14 | 83.8333 | 80.3267 | 79.1667 | 75.0667 |
| orange | 68.1767 | 66.6 | 22.9667 | 67.0333 | 64.8 |
| onion | 56.1367 | 64.7333 | 26 | 31.4667 | 28.2 |
| opuntia | 58.5 | 10.3333 | 82.4667 | 37.6667 | 33 |
| pomegranate | 63.6667 | 59.3333 | 88.6667 | 59 | 41.3333 |

|  | Pb | Cu | Cr | Zn | Se |
| --- | --- | --- | --- | --- | --- |
| banana | 82.5333 | 75.1667 | 52.5 | 71.3667 | 70.4667 |
| potato | 99.8333 | 99.5 | 99.7333 | 99.6 | 99.5333 |
| orange | 91.5333 | 89.1667 | 55.8333 | 84.8 | 84.3667 |
| onion | 81.0667 | 86.5 | 57.8333 | 58.3333 | 51.6667 |
| opuntia | 68.3667 | 17.4 | 85.8333 | 70.3333 | 52.3333 |
| pomegranate | 77 | 74.3333 | 96 | 71 | 66.3333 |

|  | Pb | Cu | Cr | Zn | Se |
| --- | --- | --- | --- | --- | --- |
| banana | 94.9333 | 92.8667 | 75.1667 | 89.2 | 87.8333 |
| potato | 100 | 100 | 100 | 100 | 100 |
| orange | 99.7833 | 99.9167 | 79.1667 | 99.85 | 99.8267 |
| onion | 95.9333 | 99.85 | 80.0333 | 80.8 | 79.9333 |
| opuntia | 77.4 | 17.2667 | 87.4 | 77.4 | 61.5667 |
| pomegranate | 89 | 88.6667 | 99.9 | 88.3333 | 84.3333 |

|  | Pb | Cu | Cr | Zn | Se |
| --- | --- | --- | --- | --- | --- |
| banana | 99.95 | 99.94 | 91.4667 | 99.9333 | 80.3667 |
| potato | 100 | 100 | 100 | 100 | 80.2 |
| orange | 100 | 100 | 92.9 | 100 | 80.4 |
| onion | 99.85 | 100 | 93.4 | 99.9167 | 78.9 |
| opuntia | 81.8 | 20 | 88.8667 | 81.9 | 66.6 |
| pomegranate | 90 | 90 | 100 | 89.6667 | 89.3333 |

**Figure 9:** Effect of adsorbents doses on Cu, Cr, Pb, Se, and Zn removal using Fe NPs: at pH, 3.0, adsorbents initial concentration 3.0 mg/l, stirring rate 150 rpm, and contact time 45 min.

|  | Cu | Cr | Zn | Se | Pb |
| --- | --- | --- | --- | --- | --- |
| banana | 31.4667 | 6.26667 | 58.1333 | 22.8333 | 32.8333 |
| potato | 69.8333 | 65.9333 | 65.8333 | 55.8133 | 82.4667 |
| orange | 52.5 | 8.5 | 49.1333 | 41.4667 | 52.4667 |
| onion | 51.4667 | 12.8333 | 12.8233 | 10.3667 | 36.7667 |
| opuntia | 2.66667 | 37.3333 | 22.3333 | 8 | 36 |
| pomegranate | 55 | 59.3333 | 48.6667 | 18.6667 | 52.3333 |

|  | Cu | Cr | Zn | Se | Pb |
| --- | --- | --- | --- | --- | --- |
| banana | 68.4 | 41.4667 | 55.8333 | 54.3667 | 79.1667 |
| potato | 99.25 | 98.1333 | 97.3 | 96.7333 | 99.48 |
| orange | 81.0333 | 48.1333 | 84.8 | 83.7 | 84.8 |
| onion | 79.1667 | 45.8333 | 41.9667 | 38.2667 | 70.5 |
| opuntia | 10 | 66 | 59 | 34 | 62.6667 |
| pomegranate | 72.2 | 78.3333 | 70.3333 | 51.6667 | 68.9 |

|  | Cr | Cu | Zn | Se | Pb |
| --- | --- | --- | --- | --- | --- |
| banana | 75.1667 | 92.8667 | 89.2 | 87.8333 | 94.9333 |
| potato | 100 | 100 | 100 | 100 | 100 |
| orange | 79.1667 | 99.9167 | 99.85 | 99.8267 | 99.7833 |
| onion | 80.0333 | 99.85 | 80.8 | 79.9333 | 95.9333 |
| opuntia | 87.4 | 17.2667 | 77.4 | 61.5667 | 77.4 |
| pomegranate | 99.9 | 88.6667 | 88.3333 | 84.3333 | 89 |

|  | Cu | Cr | Zn | Se | Pb |
| --- | --- | --- | --- | --- | --- |
| banana | 89.6 | 74.0333 | 86.7 | 80.3667 | 94.7 |
| potato | 99.93 | 99.8633 | 99.7967 | 99.9167 | 99.8333 |
| orange | 99.8267 | 78.1667 | 99.7033 | 99.6733 | 99.6 |
| onion | 99.5333 | 77.9 | 79.1667 | 78.9 | 94.9 |
| opuntia | 32 | 91.6667 | 85 | 65.6667 | 92.3333 |
| pomegranate | 96.6667 | 99.9667 | 95 | 93.6667 | 96.7333 |

**Figure 8:** Influence of contact time on Cu, Cr, Pb, Se, and Zn removal using Fe NPs: at pH, 3.0, adsorbents initial concentration 3.0 mg/l, adsorbents doses 0.3 g, stirring rate 150 rpm.
