# Supplementary material for: Adsorption of Chromium, Copper, Lead, Selenium, and Zinc ions into ecofriendly synthesized magnetic iron nanoparticles
Source: PLoS One. 2023 Oct 18;18(10):e0289709. doi: 10.1371/journal.pone.0289709 (PMC10584173; doi:10.1371/journal.pone.0289709)
Supplement: Attachment — Submitted filename: response 30-6 .docx [file pone.0289709.s005.docx]

Dear Valued Editor,

Thank you for your help and support. Your comments are helpful and improving the article a lot. The following Table shows the response to these comments.

| 1 | The comments about quantitative data and conclusion in the abstract were not addressed. |  |
| --- | --- | --- |
| 2 | The Ce in the abstract is 1.0 mg/L, in the conclusion is 3.0 mg/L and in the authors response narrative is 6.0 mg/L. So which is which? | 3.0 mg/L  modified |
| 3 | Section 2.4, what is qmax | q_e_ (mg/g) modified |
| 4 | Section 2.4.1, Equation 3 is incorrect | Done - delete |
| 5 | The K_L_ and K_F_ values of the Langmuir and Freundlich must be properly addressed, not loosely as K. | Done |
| 6 | Table 1 title, that is XRF data not XRD | Done  **Table 1:** The XRF analysis of Fe-NPs (%) |
| 7 | Table 2, the data shown there is incorrect. | Done modified  **Table 2:** The Fe-NPs adsorbent's maximal adsorption capacity is compared to that of other adsorbents used in the removal of metal ions |
| 8 | Table 3 and 4, why only show R^2^ values? Why not other parameters are not shown? Consider putting other data in the supporting document. | Done  **Table 3: The parameters of Langmuir and Freundlich isotherm models for Cu, Se, Zn, Pb, and Cr adsorption on the** banana, potato, orange, onion, opuntia, and pomegranate  **Table 4:** The parameters of pseudo-second-order model parameters of Cu, Se, Zn, Pb, and Cr adsorption onto iron nanoparticles of banana, potato, orange, onion, opuntia, and pomegranate |
| 9 | Section 3.5 does not have data and it should be omitted and reported as such that reusability studies were not done. Also, in the authors comments, it is suggested repeatability was done 5.72 times. How is this possible? Fraction? | Done – delete |
| 10 | Table 6 should compare the adsorption capacity of the current study to literature not just listing the literature values alone. | Table 6 was deleted and corporated into Table 2 |
| 11 | What significant FTIR peaks were observed? This can be used to suggest successful synthesis and/ or adsorption. This value (s) can be quoted in the conclusion and abstract. | Done  The functional groups were examined and responsible for adsorption process by nanoparticle powder sample, these peaks are 3400 cm^−1^, 2900 cm^-1^, 1600 cm^−1^,1000 cm^−1^, and 1550 cm^−1^. |
| 12 | Again, what properties of Fe-NPs were explored or came to the fore during adsorption? This was asked before by a reviewer and needs to be included in the conclusion. | Done  The preparation of iron nanoparticles was characterized many distinct magnetic properties, the ability to control their size, morphology, and high surface area properties, have many functions group responsible for adsorption process |
| 13 | 13) Authors are advised to scrutinize their manuscript even for comments not made here and make necessary adjustments. | Done |

**Table 1:** The XRF analysis of Fe-NPs (%)

| **Component** | **Fe-NPs**  **banana** | **Fe-NPs Potato** | **Fe-NPs Onion** | **Fe-NPs Orange** | **Fe-NPs**  **Opuntia** | **Fe-NPs**  **Pomegranate** |
| --- | --- | --- | --- | --- | --- | --- |
| Fe_2_O_3_ | 58.9 | 53.9 | 67.3 | 46.6 | 49 | 46.6 |
| Na_2_O | 14 | 16.1 | 8.39 | 22.1 | 20.1 | 22.1 |
| MnO | 0.46 | 0.53 | 0.63 | 0.46 | 0.46 | 0.46 |
| CaO | 0.28 | 0.3 | 0.47 | 0.27 | 0.27 | 0.27 |
| SiO_2_ | 0.25 | 0.37 | 0.38 | 0.36 | 0.36 | 0.36 |
| Al_2_O_3_ | 0.1 | 0.09 | 0.08 | 0.07 | 0.07 | 0.07 |
| K_2_O | 0.05 | 0.05 | 0.14 | ---- | 0.07 | 0.07 |
| SO_3_^--^ | 0.08 | 0.09 | 0.05 | 0.07 | ---- | ---- |
| Cr_2_O_3_ | 0.05 | 0.05 | 0.05 | ---- | 0.04 | 0.04 |
| MgO | 0.04 | 0.04 | 0.04 | 0.04 | ----- | ----- |
| Cl^-^ | 16.1 | 9.63 | 11.6 | 14.9 | 13.9 | 14.9 |
| LOI | 9.61 | 18.8 | 9.51 | 15.1 | 15.1 | 15.1 |
| Total | 99.94 | 99.98 | 99.94 | 99.99 | 99.37 | 99.99 |

**Table 2:** The Fe-NPs adsorbent's maximal adsorption capacity is compared to that of other adsorbents used in the removal of metal ions

| **Adsorbents** | **Adsorbants** | **Q max** | **Reference** |
| --- | --- | --- | --- |
| **Untreated rice husk** | Direct dyes | 2.4 | (Abdelwahab, El Nemr et al. 2005) |
| **Activated rice husk** | Direct dyes | 4.3 | (Abdelwahab, El Nemr et al. 2005) |
| **Red-mud** | Ni^+2^ | 0.0018 | (Lakshmi Narayan, Govindan et al. 2013) |
| **Peanut Hulls** | Fe^+3^ and Cu^+2^ | 79.28 and 96.58 mg/g for Fe^+3^ and Cu^+^ | (Salam, Reiad et al. 2011) |
| **Zeolite derived from fly ash** | Cu^+2^ | 14.7 | (Mishra and Tiwari 2006) |
| **Ag nanoparticle-loaded activated carbon (Ag-NP-AC)** | Cu^+2^ | 60 | (Shahamirifard, Ghaedi et al. 2016) |
| **Iron oxide coated eggshell powder** | Cu^+2^ | 6.7 | (Liu, Liang et al. 2005) |
| **Chitosan** | Cu^+2^ | 62.4 | (Crini 2005) |

|  |  |
| --- | --- |
|  | |

**Figure 11: Effect of pH on removal of Cu, Pb, Se, Zn, and Cr by iron nanoparticles of banana, potato, orange, onion, opuntia, and pomegranate, the other conditions kept constant, the during the adsorption processes dose=04 g/L, Initial conc.= 3.0 mg/L and rpm 150/45 min**

**3.3 Adsorption Isotherms**

The adsorption isotherms of the studied metals on the iron nanoparticles for extracts of banana, potato, orange, onion, opuntia, and pomegranate, were based on the optimum operating conditions which were 0.4 g at pH 6.0 Figure 12. The linearization was performed according to the mathematical models of Freundlich. Table 3 shows the parameters of Langmuir and Freundlich models obtained and the correlation coefficients of adsorption data. The experimental results of Cu, Pb, Cr, Zn, and Se adsorption on the banana, potato, orange, onion, opuntia, and pomegranate comply with the Freundlich isotherm model according to R^2^ studied (Gonçalves Junior, Meneghel et al. 2013)**.** The model that best fitted for metal adsorption was Freundlich, indicating that the adsorption occurred in multiple layers(Gonçalves Junior, Meneghel et al. 2013)**.**

The calculated parameters for the Langmuir and Freundlich models are shown in Table 3, along with the correlation coefficients for the adsorption data. According to the experimental results of Cu, Pb, Cr, Zn, and Se adsorption on the iron nanoparticles of banana, potato, orange, onion, opuntia, and pomegranate, the adsorption took place in multiple layers and was consistent with the Freundlich isotherm model (Gonçalves Junior, Meneghel et al. 2013). The metals Cu, Cr, Se, and Zn have higher adsorption capacities (q_m_) and the highest binding energies with the adsorbent in the Langmuir linearization. As the adsorbents ranged from 1.3 to 388 (mg/g), the values of K_F_ for Cu, Se, Zn, Pb, and Cr were calculated from Table 3. The characteristics of each metal and the manner of interaction with the adsorbents can be linked to this adsorption sequence. The magnitudes of K_F_ demonstrate the simple removal of metal ions from the aqueous solution and suggest an advantageous adsorption process (Kumar and Kirthika 2009, Gonçalves Junior, Meneghel et al. 2013, dos Santos, de Toledo Gomes et al. 2019).

|  |  |
| --- | --- |
|  |  |
|  |  |
|  |  |
|  |  |

**Figure 12: The Langmuir and Freundlich isotherms models of Pb; Cr Se Cu, and Zn onto iron nanoparticles of banana, potato, orange, onion, opuntia, and pomegranate, the other conditions kept constant, the during the adsorption processes dose=04 g/L, and rpm 150/45 min**

**Table 3:** **The Langmuir and Freundlich isotherm models for Cu, Se, Zn, Pb, and Cr adsorption on the banana, potato, orange, onion, opuntia, and pomegranate**

| **Langmuir isotherm models** | **R_2_** | | | | | **q_max_** | | | | | **K_L_** | | | | |
| --- | --- | --- | --- | --- | --- | --- | --- | --- | --- | --- | --- | --- | --- | --- | --- |
|  | **Pb** | **Cu** | **Cr** | **Zn** | **Se** | **Pb** | **Cu** | **Cr** | **Zn** | **Se** | **Pb** | **Cu** | **Cr** | **Zn** | **Se** |
| **Fe NPs (Banana)** | **0.7345** | **0.7767** | **0.7954** | **0.8237** | **0.9674** | **25000** | **33333** | **33333** | **23** | **22** | **0.0002** | **0.66** | **0.76** | **0.73** | **0.75** |
| **Fe NPs (Potato)** | **0.4878** | **0.9621** | **0.9621** | **0.4749** | **0.4709** | **25000** | **33653** | **33245** | **49595** | **50000** | **0.0003** | **0.0002** | **0.0002** | **0.0002** | **0.0002** |
| **Fe NPs (Orange)** | **0.6942** | **0.6439** | **0.6436** | **0.6955** | **0.6936** | **33333** | **33457** | **34873** | **34332** | **33333** | **0.0002** | **0.0002** | **0.0002** | **0.0002** | **0.0002** |
| **Fe NPs (Onion)** | **0.8393** | **0.6949** | **0.6949** | **0.8667** | **0.0017** | **25000** | **34233** | **33333** | **21** | **20** | **0.0002** | **0.0014** | **0.13** | **0.16** | **0.81** |
| **Fe NPs (Opuntia)** | **0.3581** | **0.2771** | **0.2778** | **0.9704** | **0.9478** | **20000** | **44** | **43** | **20** | **20** | **0.0006** | **0.0005** | **0.13** | **0.98** | **1.0** |
| **Fe NPs (Pomegranate)** | **0.964** | **0.9661** | **0.966** | **0.941** | **0.9869** | **58** | **55** | **58** | **21** | **22** | **0.18** | **0.16** | **0.18** | **0.64** | **0.62** |
|  | **R^2^** | | | | | **N** | | | | | **K_F_** | | | | |
| **Freundlich isotherm models** | **Pb** | **Cu** | **Cr** | **Zn** | **Se** | **Pb** | **Cu** | **Cr** | **Zn** | **Se** | **Pb** | **Cu** | **Cr** | **Zn** | **Se** |
| **Fe NPs (Banana)** | **0.8554** | **0.9791** | **0.9792** | **0.9056** | **0.9789** | **5.53** | **5.33** | **6.33** | **7.01** | **7.22** | **9.0** | **9.40** | **9.13** | **8.7** | **6.5** |
| **Fe NPs (Potato)** | **0.889** | **0.977** | **0.9873** | **0.7134** | **0.7042** | **0.24** | **0.34** | **0.42** | **0.98** | **1.55** | **343** | **12.3** | **66** | **54** | **44** |
| **Fe NPs (Orange)** | **0.876** | **0.8702** | **0.8679** | **0.8701** | **0.8661** | **0.21** | **0.33** | **0.54** | **6.98** | **6.87** | **345** | **12.3** | **22** | **14** | **13** |
| **Fe NPs (Onion)** | **0.9449** | **0.8722** | **0.8679** | **0.9332** | **0.1545** | **0.20** | **6.8** | **7.40** | **7.35** | **7.44** | **388** | **12.3** | **55** | **14** | **12** |
| **Fe NPs (Opuntia)** | **0.9757** | **0.9772** | **0.4986** | **0.988** | **0.9767** | **0.64** | **4.4** | **4.46** | **3.65** | **3.98** | **55** | **1.3** | **1.3** | **1.5** | **1.3** |
| **Fe NPs (Pomegranate)** | **0.9831** | **0.9775** | **0.9725** | **0.9726** | **0.9961** | **0.59** | **1.7** | **1.72** | **1.56** | **1.66** | **66** | **13** | **11** | **1.4** | **11** |

**3.4. Kinetic study**

Figure 13 shows the kinetics models for Cu, Pb, Se, Zn, and Cr ion adsorption onto banana, potato, orange, onion, opuntia, and pomegranate. The relation between log (q_eq-_q_t_) and t. The K_1_ and q_e_ are obtained from the slope and intercept, respectively. The correlation coefficients (R^2^) of the pseudo-first-order kinetic model are non-linear but the pseudo-second-order kinetic model linear line, the correlation coefficients (R^2^) of the pseudo-second-order is shown in **Table 4:**. The results suggested that the overall rates of adsorption of Cu, Pb, Se, Zn, and Cr ions onto banana, potato, orange, onion, opuntia, and pomegranate were controlled by chemisorption (Ibrahim Hegazy 2021). Besides, the value of R^2^ (linear correlation) around 1 in pseudo-second-order confirms that the adsorption kinetics is controlled by this order and that there is a strong interaction between adsorbent and adsorbate.

Figures 13 showed the kinetics models for Cu, Pb, Se, Zn, and Cr ion adsorption onto banana, potato, orange, onion, opuntia, and pomegranate. The pseudo-first-order kinetic model shows in Figure 13, that’s shows the relation between log (q_eq-_q_t_) and t, and through this relation the values K_1_ and q_e_ could be deduced from the slope and intercept, respectively. The correlation coefficients (R^2^) of the pseudo-second-order kinetic model are higher than the pseudo-first-order kinetic model, and q_e_ values calculated from the pseudo-second-order kinetic model are very close to the experimental with freundlich isotherm model. The results suggested that the overall rates of adsorption of Cu, Pb, Se, Zn, and Cr ion onto adsorption onto banana, potato, orange, onion, opuntia, and pomegranate by physical adsorption [46].

**Figure 13:** The pseudo-first-order and pseudo-second-order of Pb, Cr, Se, Cu, and Zn onto iron nanoparticles of banana, potato, orange, onion, opuntia, and pomegranate, the other conditions kept constant, the during the adsorption processes dose=04 g/L, initial concentration 3.0 mg/L, and speed 150 rpm

**Table 4:** The parameters of pseudo-second-order model parameters of Cu, Se, Zn, Pb, and Cr adsorption onto iron nanoparticles of banana, potato, orange, onion, opuntia, and pomegranate

| **Adsorbents/ metal** | R^2^ | | | | | **q** | | | | | **K_2_** | | | | |
| --- | --- | --- | --- | --- | --- | --- | --- | --- | --- | --- | --- | --- | --- | --- | --- |
|  | **Pb** | **Cu** | **Cr** | **Zn** | **Se** | **Pb** | **Cu** | **Cr** | **Zn** | **Se** | **Pb** | **Cu** | **Cr** | **Zn** | **Se** |
| **FeNPs/Potato** | 0.9974 | 0.9974 | 0.9974 | 0.9134 | 0.9001 | 10.8 | 10.75 | 7.14 | 7.14 | 7.78 | 0.001 | 0.001 | 0.015 | 0.01 | 0.01 |
| **FeNPs/Banana** | 0.9425 | 0.9712 | 0.9567 | 0.9223 | 0.9113 | 10.43 | 10.30 | 1.88 | 1.88 | 1.98 | 0.001 | 0.001 | 0.007 | 0.007 | 0.007 |
| **FeNPs/Onion** | 0.9325 | 0.9711 | 0.225 | 0.9012 | 0.913 | 9.11 | 9.01 | 8.33 | 8.33 | 8.76 | 0.003 | 0.004 | 0.0008 | 0.0008 | 0.0007 |
| **FeNPs/Opuntia** | 0.9911 | 0.9468 | 0.9911 | 0.915 | 0.9563 | 0.87 | 0.83 | 11.11 | 100 | 101 | 0.011 | 0.013 | 0.0016 | 0.00001 | 0.00001 |
| **FeNPs/Orang** | 0.9971 | 0.9717 | 0.9442 | 0.9334 | 0.9553 | 11.28 | 11.23 | 3.03 | 3.03 | 3.12 | 0.001 | 0.001 | 0.003 | 0.003 | 0.004 |
| **FeNPs/pomegranate** | 0.9974 | 0.9525 | 0.9627 | 0.9011 | 0.991 | 11.87 | 11.36 | 8.33 | 8.33 | 8.34 | 0.001 | 0.0015 | 0.005 | 0.003 | 0.003 |
